# Nanosecond resistive switching in Ag/AgI/PtIr nanojunctions

**DOI:** 10.3762/bjnano.11.9

**Published:** 2020-01-08

**Authors:** Botond Sánta, Dániel Molnár, Patrick Haiber, Agnes Gubicza, Edit Szilágyi, Zsolt Zolnai, András Halbritter, Miklós Csontos

**Affiliations:** 1 Department of Physics, Budapest University of Technology and Economics, Budafoki út 8, H-1111 Budapest, Hungary; 2 MTA-BME Condensed Matter Research Group, Budafoki út 8, H-1111 Budapest, Hungary; 3 Department of Physics, University of Konstanz, Universitätstrasse 10, D-78464 Konstanz, Germany; 4 Empa, Swiss Federal Laboratories for Materials Science and Technology, Transport at Nanoscale Interfaces Laboratory, Überlandstrasse 129, CH-8600 Dübendorf, Switzerland; 5 Wigner Research Centre for Physics, Hungarian Academy of Sciences, Konkoly-Thege Miklós út 29-33, H-1121 Budapest, Hungary; 6 Centre for Energy Research, Institute of Technical Physics and Materials Science, Hungarian Academy of Sciences, Konkoly-Thege Miklós út 29-33, H-1121 Budapest, Hungary

**Keywords:** memristor, nanojunction, nanosecond operation, resistive switching, silver iodide (AgI)

## Abstract

Nanometer-scale resistive switching devices operated in the metallic conductance regime offer ultimately scalable and widely reconfigurable hardware elements for novel in-memory and neuromorphic computing architectures. Moreover, they exhibit high operation speed at low power arising from the ease of the electric-field-driven redistribution of only a small amount of highly mobile ionic species upon resistive switching. We investigate the memristive behavior of a so-far less explored representative of this class, the Ag/AgI material system in a point contact arrangement established by the conducting PtIr tip of a scanning probe microscope. We demonstrate stable resistive switching duty cycles and investigate the dynamical aspects of non-volatile operation in detail. The high-speed switching capabilities are explored by a custom-designed microwave setup that enables time-resolved studies of subsequent set and reset transitions upon biasing the Ag/AgI/PtIr nanojunctions with sub-nanosecond voltage pulses. Our results demonstrate the potential of Ag-based filamentary memristive nanodevices to serve as the hardware elements in high-speed neuromorphic circuits.

## Introduction

The half a century long increase of the computational capacity of von Neumann architectures built on ever-shrinking complementary metal-oxide semiconductor (CMOS)-based hardware units is facing its technological, economical and fundamental barriers: (i) Further miniaturization of the CMOS transistors below 10 nm channel length is technologically extremely demanding and cost-inefficient. (ii) Their digital operation is not sustainable below the scale of the Fermi wavelength, which is typically ca 10 nm in layered semiconductors. (iii) Moreover, the so-called von Neumann bottleneck, i.e., the bandwidth-limited and power-hungry permanent data transfer between the physically separated processing, memory and permanent storage units prevents the large-scale establishment of autonomous, energy-efficient “internet of things (IoT)” hardware solutions [1].

Two-terminal, non-volatile resistance-change memory devices (RRAMs) [2–4], the operation of which relies on controllable, electric-field-induced structural changes in an electronically insulating ionic conductor medium, offer a viable alternative to intrinsically overcome the above limitations: (i) Due to their self-assembled, filamentary nature, the macroscopically observable conductance features of the devices are determined by lithographically inaccessible, metallic volumes close to the atomic scale. (ii) The Fermi wavelength in these filaments falls in the regime of the interatomic distances granting metallic conductance also in this ultimate scaling limit. (iii) The device conductance is largely determined by the rearrangement of only a few atoms in this narrowest cross section, which can take place at a very large bandwidth and unprecedentedly low energy cost [5–9]. Furthermore, since such rearrangements are subject to activated processes, the induced conductance changes are typically non-volatile at sub-threshold electric fields. As a consequence, networks of filamentary RRAM devices naturally lend themselves for the realization of physically unified platforms for all tasks of information processing. As an example, large crossbar arrays of transition-metal-oxide-based filamentary RRAM devices operated in the metallic conductance regime of 10^2^–10^4^Ω have been successfully utilized recently to perform various linear operations relying on hardware-implemented vector–matrix multiplication [10–12]. An access to a continuum of resistance states exhibiting highly linear current–voltage [*I*(*V*)] characteristics were exploited to achieve a numerical precision up to 8 bits.

Ag-based filamentary resistive switches [13–27] have been promoted in terms of the “atomic switch” by K. Terabe et al. utilizing Ag_2_S as the ionic conductor medium [28]. The roles of the matrix, the electrode, and their interfaces in the filament formation have been extensively investigated covering a broad range of materials systems [4]. Although AgI has been recognized as an intrinsic ionic conductor supporting high Ag^+^ ionic mobilities already since the pioneering era of photography, thorough studies exploring its resistive switching properties are still relatively under-represented in the literature [29–34]. In this paper we explore the dynamical properties of room-temperature resistive switching established in metallic Ag/AgI/PtIr nanojunctions. Hysteretic *I*(*V*) traces were studied as a function of the frequency and the amplitude of triangular voltage signals covering five orders of magnitude in the time domain testifying to the voltage–time dilemma [22,35] commonly observed in Ag-based filamentary resistive switches. In order to challenge device operation in AgI-based nanoswitches down to sub-nanosecond timescales for the first time, a special purpose pulsed microwave setup was developed and successfully utilized to fire 500 ps long set/reset voltage pulses and acquire the resulting resistive switching at 1 GHz bandwidth.

## Results and Discussion

### Structural and electrical characterization

**Figure 1:**
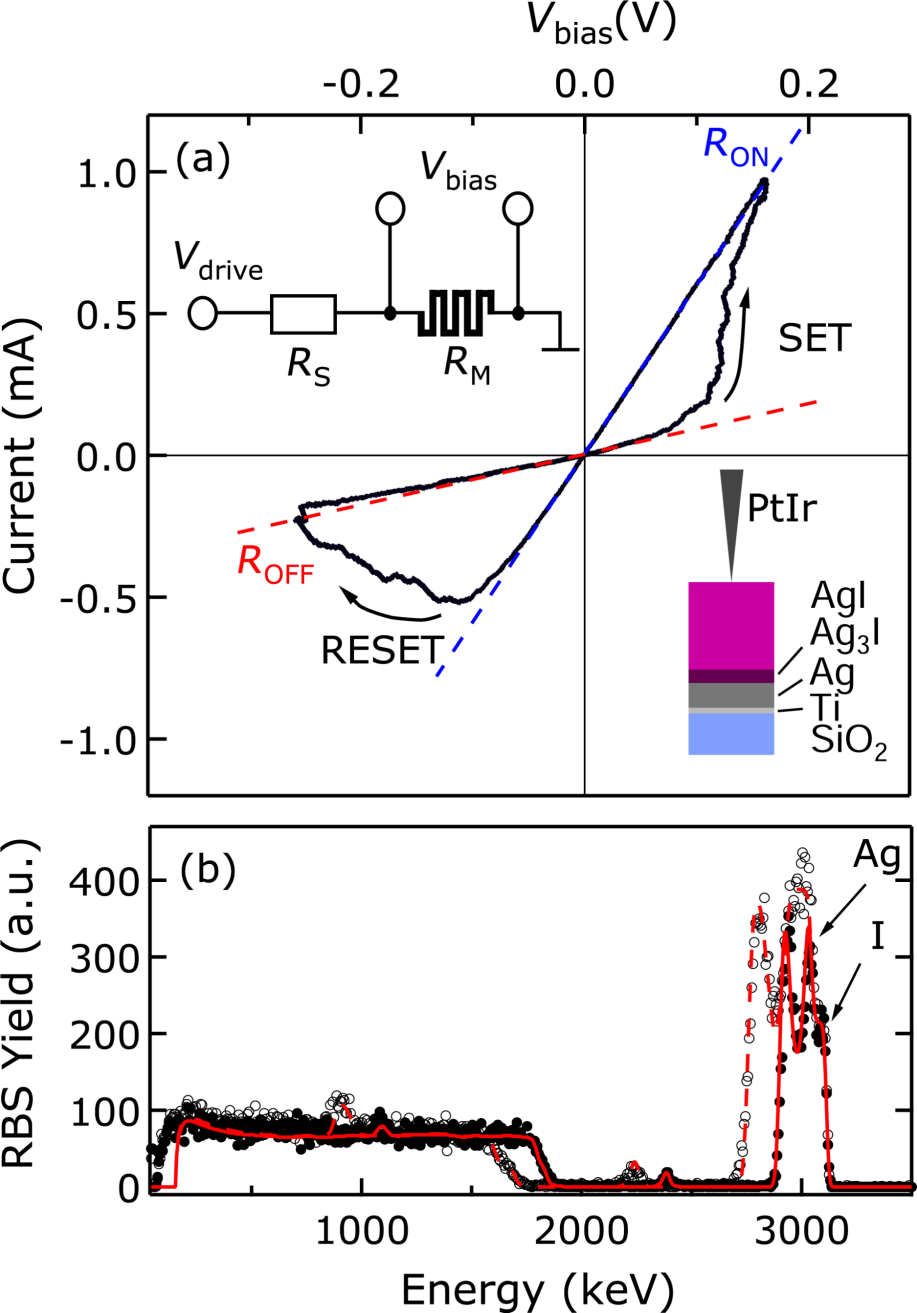
(a) Representative *I*(*V*) trace of an Ag/AgI/PtIr nanojunction demonstrating resistive switching behavior at room temperature. The *R*_OFF_ and *R*_ON_ OFF and ON state resistances are evaluated from the corresponding zero-bias slopes as indicated by the red and blue dashed lines, respectively. *R*_S_ = 150 Ω. The lower inset illustrates the sample structure. The upper inset shows the dc measurement setup. (b) Energy-resolved RBS spectra of a 200 nm thick AgI thin film recorded at 7° (full circles) and 60° (empty circles) tilt angles. The red solid and dashed lines display the corresponding model spectra.

Memristive nanojunctions were created by approaching a mechanically sharpened PtIr tip of a custom-built scanning tunneling microscope (STM) to the AgI-coated thin film structure schematically illustrated in the lower inset of Figure 1a.

**Figure 2:**
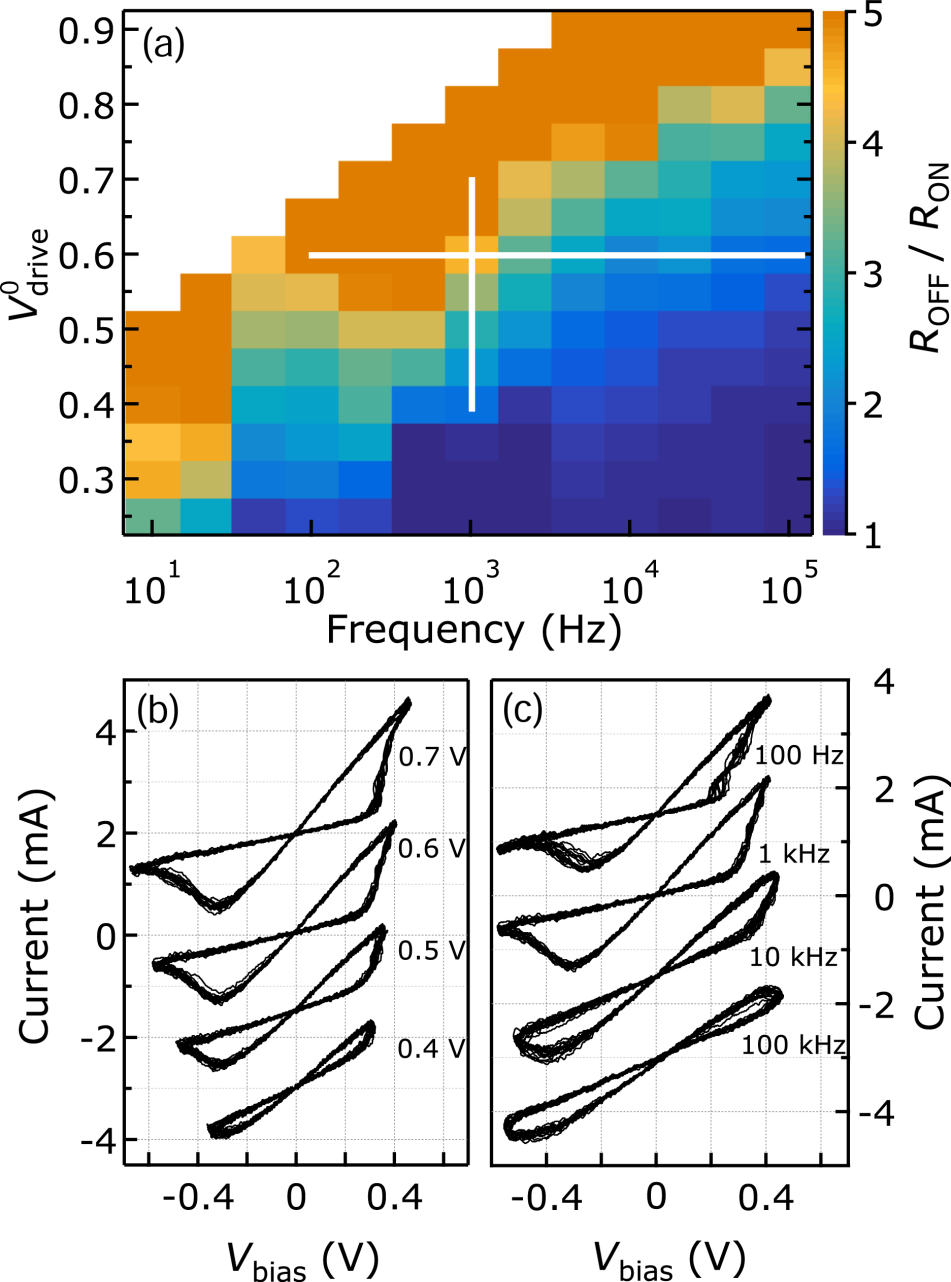
(a) The color scale shows the OFF-to-ON-resistance ratio, *R*_OFF_/*R*_ON_, of a stable Ag/AgI/PtIr nanojunction as a function of the frequency *f*_drive_ and the amplitude 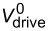 of the triangular driving voltage signal. The vertical and horizontal white lines correspond to the regime of the data selected in panels (b) and (c), respectively. (b) Representative hysteretic *I*(*V*) traces recorded at *f*_drive_ = 1 kHz and different 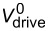 amplitudes. (c) Representative hysteretic *I*(*V*) traces recorded at 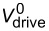 = 0.6 V and different values of *f*_drive_. 10 consecutive cycles were recorded at each driving parameter set. *R*_S_ = 150 Ω. The *I*(*V*) traces of panels (b) and (c) are off-set along the current axes for clarity.

The photosensitive AgI layers were formed promptly before electrical characterization by exposing a 100 nm thick Ag layer to iodine vapor at 40°C and ambient pressure for 30 s in the dark following the method of Kumar and co-workers [36]. The Ag films were evaporated on standard Si/SiO_2_ wafers using a 12 nm thick Ti sticking layer. The structural characterization of the thin film samples was carried out by Rutherford backscattering spectrometry (RBS) using an ion beam of 3500 keV ^4^He^+^ hitting the sample surface at various incident angles at a base pressure of 10^−4^ Pa. The angle-dependent energy spectra of the backscattered He^+^ ions were recorded and evaluated by comparing to theoretical spectra calculated using RBX [37] as shown in Figure 1b. This comparative analysis revealed a layer structure (top to bottom) of 200 nm AgI, 22.5 nm Ag_3_I and 43 nm Ag after iodine exposure on the Ti/SiO_2_/Si substrate. Nanometer-scale Ag/AgI/PtIr nanojunctions were created by bringing the PtIr tip into direct contact with the thin-film surface while a constant bias voltage of 100 mV was applied on the former. Preset contact resistance values of a few kiloohms were achieved by employing a current-controlled feedback loop. The first few periods of subsequent *I*(*V*) measurements were dominated by unstable, non-memristive curves, attributed to the initial formation of the metallic filament. They were directly followed by the onset of stable hysteretic traces without the need of any further dedicated electroforming procedure. We argue, that the indention of the tip to the surface layer reduces the effective thickness of the dielectric layer resulting in the down-scaling of the electroforming voltage to the range of the set voltage in agreement with the tendency commonly observed in nanoscale resistive switching systems [8–9,22]. Resistive switching was characterized by the analysis of hysteretic *I*(*V*) traces that were acquired by using the setup shown in the upper inset of Figure 1a. A 2.5 Hz triangular driving voltage signal, *V*_drive_, was applied to the memristive junction and a series resistor with corresponding resistances of *R*_M_ and *R*_S_, respectively. Typical values of *R*_S_ were in the range of 50–1000 Ω. The current was monitored by a current amplifier whereas the *V*_bias_ voltage drop on the memristor was determined numerically as *V*_bias_ = *V*_drive_ − *I*·*R*_S_. As a polarity convention, a positive bias corresponds to a higher potential applied on the planar Ag electrode with respect to the PtIr tip. A representative *I*(*V*) trace acquired within 400 ms is exemplified in Figure 1a. Bipolar resistive switching takes place when the magnitude of *V*_bias_ exceeds approx. 100 mV at either polarity. The minor deviation of the OFF and ON state *I*(*V*) branches from their zero-bias slopes (red and blue dashed lines, respectively) up to the switching threshold voltages is a hallmark of metallic conduction indicating the presence of continuous filaments in both states. At positive voltage the presence of the series resistor is considered as restrictive factor for further resistance change, whereas at negative voltage it is the saturation with Ag^+^ ions in the junction neighborhood. These two factors allow us to keep the junction resistance in the targeted metallic resistance range, where the nonlinear tunneling *I*(*V*) characteristics, which are unfavorable for neuromorphic operations, are avoided. These characteristics were further investigated as a function of the amplitude 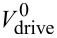 and the frequency *f*_drive_ of the triangular driving voltage signal as shown in Figure 2.

Figure 2a summarizes the *R*_OFF_/*R*_ON_ resistance ratio values evaluated from 10^3^ individual *I*(*V*) traces acquired on a single, stable nanojunction while *f*_drive_ was varied over five orders of magnitude. We found that *R*_OFF_/*R*_ON_ linearly increases with 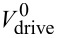 while a logarithmic decrease is observed with increasing *f*_drive_, in agreement with the so-called “voltage–time dilemma” [22,35] enabling non-volatile data storage. Exemplary traces selected from the parameter regimes highlighted in white in Figure 2a demonstrate these tendencies, as illustrated in Figure 2b and Figure 2c, where either 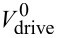 or *f*_drive_ was stepped while the other parameter was kept constant.

**Figure 3:**
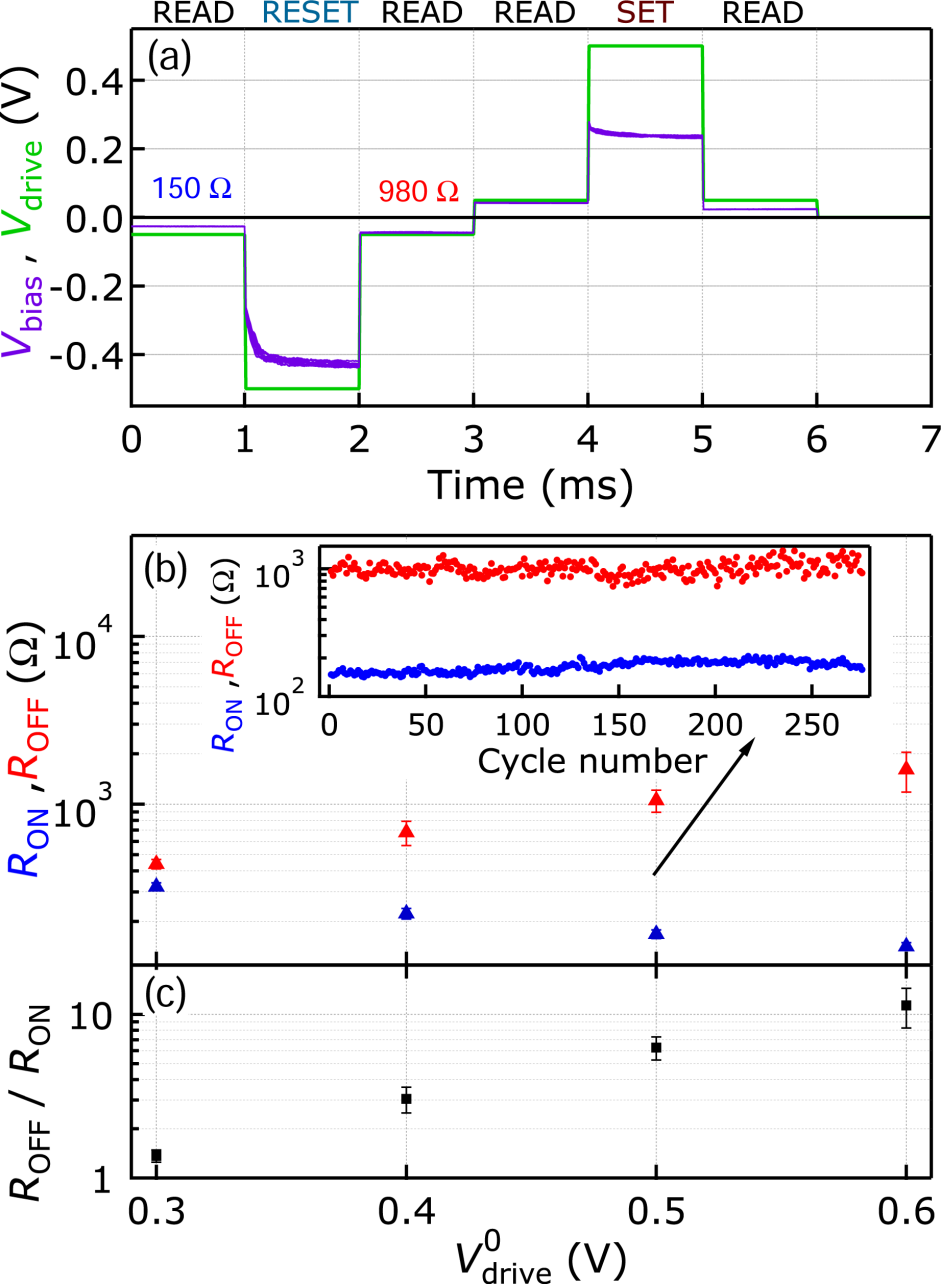
(a) A full cycle of a pulsing experiment consisting of *V*_drive_ (green) voltage pulses with ±0.5 V amplitude and 1 ms duration executing subsequent reset and set transitions. Between the large-amplitude switching pulses a ±50 mV amplitude read-out voltage was applied. The simultaneously recorded *V*_bias_ (purple) trace shows the dynamics of the resistive switchings. (b) *R*_OFF_ (red) and *R*_ON_ (blue) as a function of the 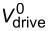 voltage pulse amplitude. The inset demonstrates the long-term stability of the OFF and ON states evaluated during 300 repetitive duty cycles. (c) The corresponding *R*_OFF_/*R*_ON_ ratio. The symbols and error bars in panels (b) and (c) correspond to the average and standard deviation of ten consecutive switching cycles. *R*_S_ = 150 Ω.

Subsequent set and reset transitions were also triggered by square-shaped *V*_drive_ voltage pulses of varying magnitude. A typical scheme utilizing 1 ms long and 0.5 V high switching pulses of alternating sign is shown by the solid green line in Figure 3a.

Between the switching pulses a constant *V*_drive_ = 50 mV was applied, also with alternating sign, to measure the actual device resistance. The independently acquired *V*_bias_ voltage signal acting on the memristive sample alone, shown in Figure 3a by the solid purple line, allowed for real-time studies of the switching dynamics. The emergence of multiple timescales governing the resistive transition is clearly seen in *V*_bias_. This is attributed to the interplay between the voltage-dividing effect of the series resistor and the exponential slow-down/speed-up of the switching at linearly varying *V*_bias_, in agreement with the data presented in Figure 2 as well as with our earlier work reported on Ag/Ag_2_S/PtIr nanojunctions [22]. The dependence of *R*_OFF_, *R*_ON_, and their ratio on the switching pulse amplitude 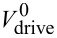 are shown in Figure 3b and Figure 3c. These reveal that the monotonous increase in the *R*_OFF_/*R*_ON_ ratio arises from the simultaneous increase (decrease) of *R*_OFF_ (*R*_ON_) as 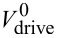 increases. Such a gradual variation for both states supports our picture of the presence of continuous metallic filaments throughout the entire investigated resistance regime. The endurance is demonstrated in the inset of Figure 3b by displaying the long-term stability of the resistance states over 300 subsequent switching cycles recorded at 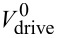 = 0.5 V. All these results demonstrate that the Ag/AgI system shows characteristics and stability very similar to the Ag/Ag_2_S system with the single exception of its photosensitivity. The latter offers a further possibility for a combined electrical and optical manipulation of the resistance states.

### Instrumental developments for high-speed resistive switching measurements

**Figure 4:**
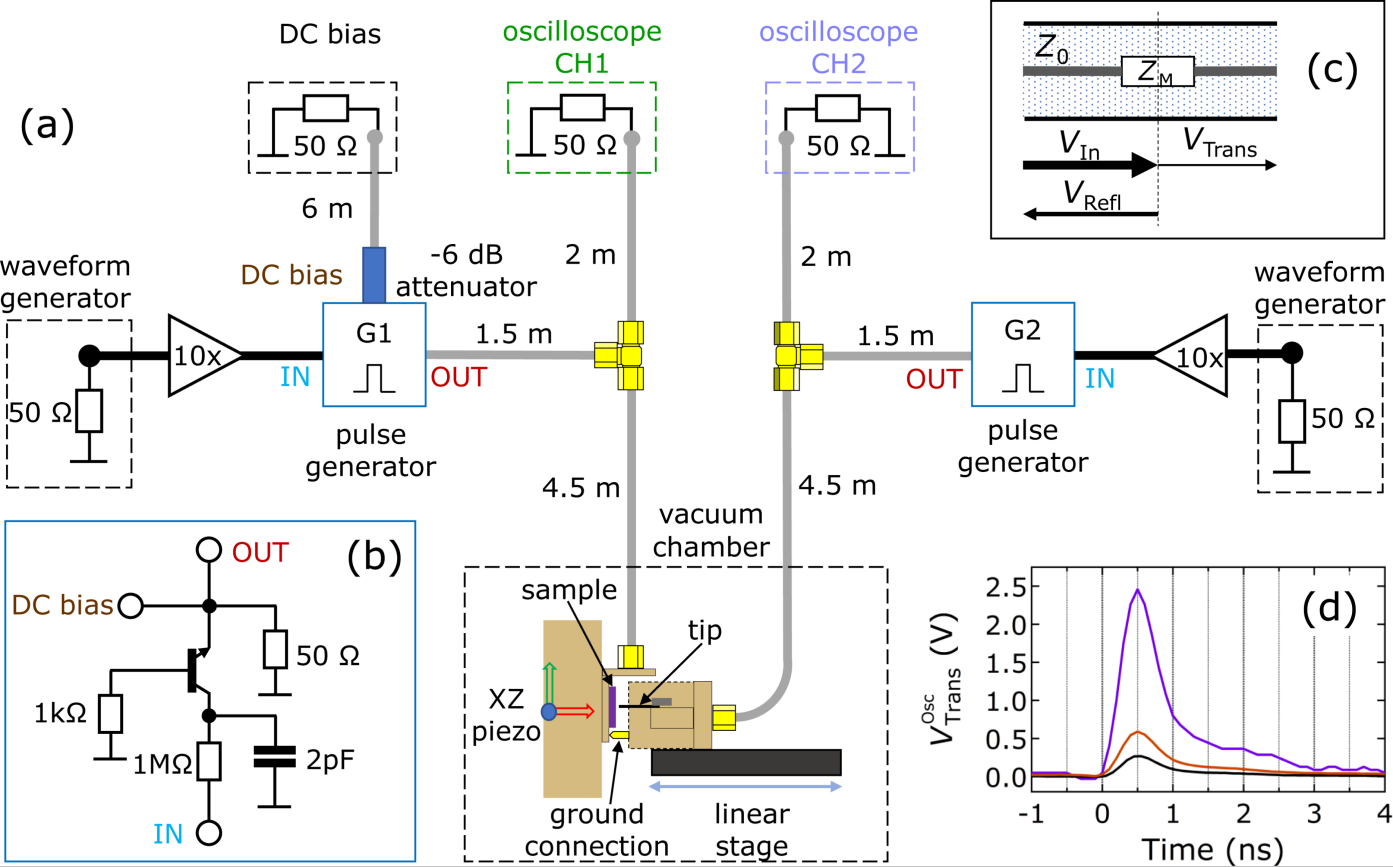
(a) The schematics of the high-frequency measurement setup. The PtIr tip and the AgI/Ag thin film are mounted on and brought in contact by individual piezo drives and a step-motor-driven linear stage. Sub-nanosecond set and reset voltage pulses are supplied by the identical avalanche pulse generators G1 and G2. Their schematics is shown in panel (b). G1 and G2 are powered by the amplified voltage signals of two identical waveform generators. The transmitted and reflected voltage signals are detected by a two-channel oscilloscope. A DC bias offset can be added to the nanojunction via the DC terminal of G1. The length of the connecting coaxial cables is indicated. (c) Scheme of our arrangement. The sample is inserted to the central wire of a coaxial cable with *Z*_0_ = 50 Ω wave impedance. The relative amplitudes of the reflected and transmitted signals are determined by the telegraph equations (see text). (d) Representative transmitted voltage pulse shapes measured by replacing the nanojunction sample with a short circuit (purple), a 330 Ω resistor (brown) and a 810 Ω resistor (black).

In order to investigate resistive switching in STM based nanojunctions during the exposure to extremely short voltage pulses of alternating sign, a special-purpose setup was designed and developed, which is described in detail in the following. Figure 4a illustrates the schematic of the setup.

In order to avoid the degradation of ambient-sensitive samples, the junctions are formed in a vacuum chamber that can be evacuated down to 10^−7^ mbar. The thin film sample and the STM tip are mechanically attached directly to the hot lines of two impedance-matched co-planar waveguide printed circuit boards (PCBs). Their common ground is provided by a spring-loaded contact, which ensures stable connection also during the mechanical adjustments of the STM junction. The PCBs hosting the STM tip and the thin film sample are mounted on a linear stage and on an *XZ* piezo actuator, respectively. The former is responsible for the coarse approach of the tip whereas the latter is used for fine adjustments as well as a lateral displacement. Both PCBs are terminated with impedance-matched SMA-type connectors certified to 0–18 GHz bandwidth. These terminals are connected to the 50 Ω matched channels of a 1 GHz bandwidth and 100 ps sampling rate digital oscilloscope, as well as to nominally identical, custom-built unipolar rise-time avalanche pulse generators [38] (G1 and G2). Subsequent set and reset transitions of the memristive nanojunctions are achieved by firing single voltage pulses from the two generators in an alternating manner. The layout of the pulse generators, which are implemented on identical, impedance-matched printed co-planar waveguide circuit boards, is shown in Figure 4b. The operation of G1 and G2 relies on the recurring charging and avalanche break-down of the 2N2369-type npn transistor. Charging occurs through the 1 MΩ resistor and the 2 pF capacitor located at the IN terminal whereas discharging takes place via the 50 Ω resistor and the load connected to the OUT terminal. The 1 kΩ resistor at the base of the transistor serves as an over-current protection. The avalanche breakdown of the bipolar transistor results in unipolar voltage pulses specified to 500 ps FWHM and, depending on the load, up to 10 V amplitude at an average repetition rate of approx. 100 kHz. The continuous pulsing operation of G1 and G2 requires the constant application of *V*_IN_ = 80 V. However, due to their stochastic nature, the timing of the avalanche events is characterized by a probability distribution rather than a well-defined repetition frequency. Our experience shows that restricting the duration of the *V*_IN_ = 80 V bias to 10 μs results in the release of a single voltage pulse. Such a driving scheme was implemented by the 10-fold amplified, slower square voltage pulse signals of the two arbitrary waveform generators (AWGs) shown in Figure 4a, operated with a suitably chosen relative timing. The AWGs were also utilized to trigger the read-out of the CH1 and CH2 oscilloscope channels. The repetition rate of the alternating set/reset transitions were limited by approx. 10 μs recovery time of the pulse generators after their avalanche breakdown.

In order to determine the resistance of the memristive junction between the switching pulses, a DC voltage offset can be applied to the junction via the DC bias terminal of G1. Taking into account the 50 Ω input impedances of the oscilloscope channels as well as the 50 Ω terminations of the OUT terminals of G1 and G2, the resistance of the junction can be calculated as *R*_M_ = (*V*_CH1_/*V*_CH2_ − 1) · 25 Ω. When the resistance of the sample does not change during the voltage pulses (e.g., when using test resistors), its value can alternatively be determined also from the magnitude of the transmitted , *V*_Trans_, and reflected, *V*_Refl_, voltage signals as described by the solution of the telegraph equations derived for the arrangement shown in Figure 4c:

[1]
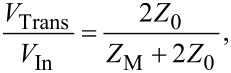


[2]
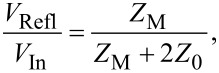


[3]
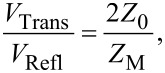


where *V*_In_ is the amplitude of the incident voltage pulse, *Z*_M_ is the impedance of the sample, and *Z*_0_ = 50 Ω is the wave impedance of the coaxial lines. Note, that as the transmitted and the reflected signals approach the oscilloscope they are attenuated through the cables (0.22 dB/m), and at the T-piece (ca. 10% of the power is reflected and the remaining power is equally split towards the oscilloscope and the pulse generators). However, the routes of the reflected and transmitted waves towards CH1 and CH2 suffer from exactly the same attenuation. Therefore the 
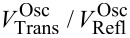
 ratio measured at the oscilloscope is the same as Equation 3, which is evaluated next to the junction. Figure 4d displays 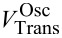 when the memristor junction is replaced by a short circuit (purple line), or when commercial surface-mounted device (smd) resistors replace the junction. The highest transmission (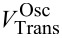 = 2.45 V peak) is recorded for the short circuit. In contrast, the 330 Ω (brown line) and 810 Ω (black line) resistors, substantially mismatching the *Z*_0_ = 50 Ω wave impedance of the connecting 20 GHz bandwidth coaxial lines, greatly suppress the transmission. This effect is in quantitative agreement with Equation 1–Equation 3. The pulse shapes of G1 and G2 are experimentally characterized based on the statistical analysis of 10000 traces to a FWHM of 514 ± 4 ps and a rise time of 274 ± 2 ps, which are close to the specified values. Figure 4d also illustrates the emergence of a slower decay at the bottom of the falling edge of the pulses. The amplitude of this tail is by a factor of five smaller than the peak height. Further care was taken to impede the effect of undesired reflections present in the circuit. These inevitably arise from even the smallest amount of imperfections in the impedance matchings at the T-junctions and at the instrument terminals. In order to be able to resolve resistive switchings during and right after the outburst of the primary voltage pulses supplied by G1 and G2 without the disturbing effects of such echo signals we shifted the latter in time by utilizing longer coaxial cables in the setup. The length of the individual segments are indicated in Figure 4a. This is an uncommon strategy in microwave technologies due to the losses introduced by the longer cables. Therefore we started our experiments by the thorough characterization of the reflections and losses imposed by the abovementioned circuit elements. Special attention was paid to the DC bias port of G1, which is directly attached to the OUT terminal of G1 and is, therefore, prone to divide and distort the output pulse shape of G1. To this end, a low-bandwidth RG178-type 6 m long coaxial cable was chosen to connect G1 and the DC bias source, exhibiting −90 dB attenuation already at 400 MHz. Inserting an additional −6 dB attenuator served to further reduce undesired reflections and, thus, maintain the symmetric microwave signal paths between the nanojunction, the pulse generators and the oscilloscope inputs.

In the following, we apply this setup to study the sub-nanosecond resistive switching in Ag/AgI/PtIr resistive switching junctions. We emphasize that this microwave arrangement is best suited to study the targeted metallic resistance range, as at higher resistances RC time-constant limitations would arise, and the transmitted signal would become unmeasurably small.

### Nanosecond resistive switchings

**Figure 5:**
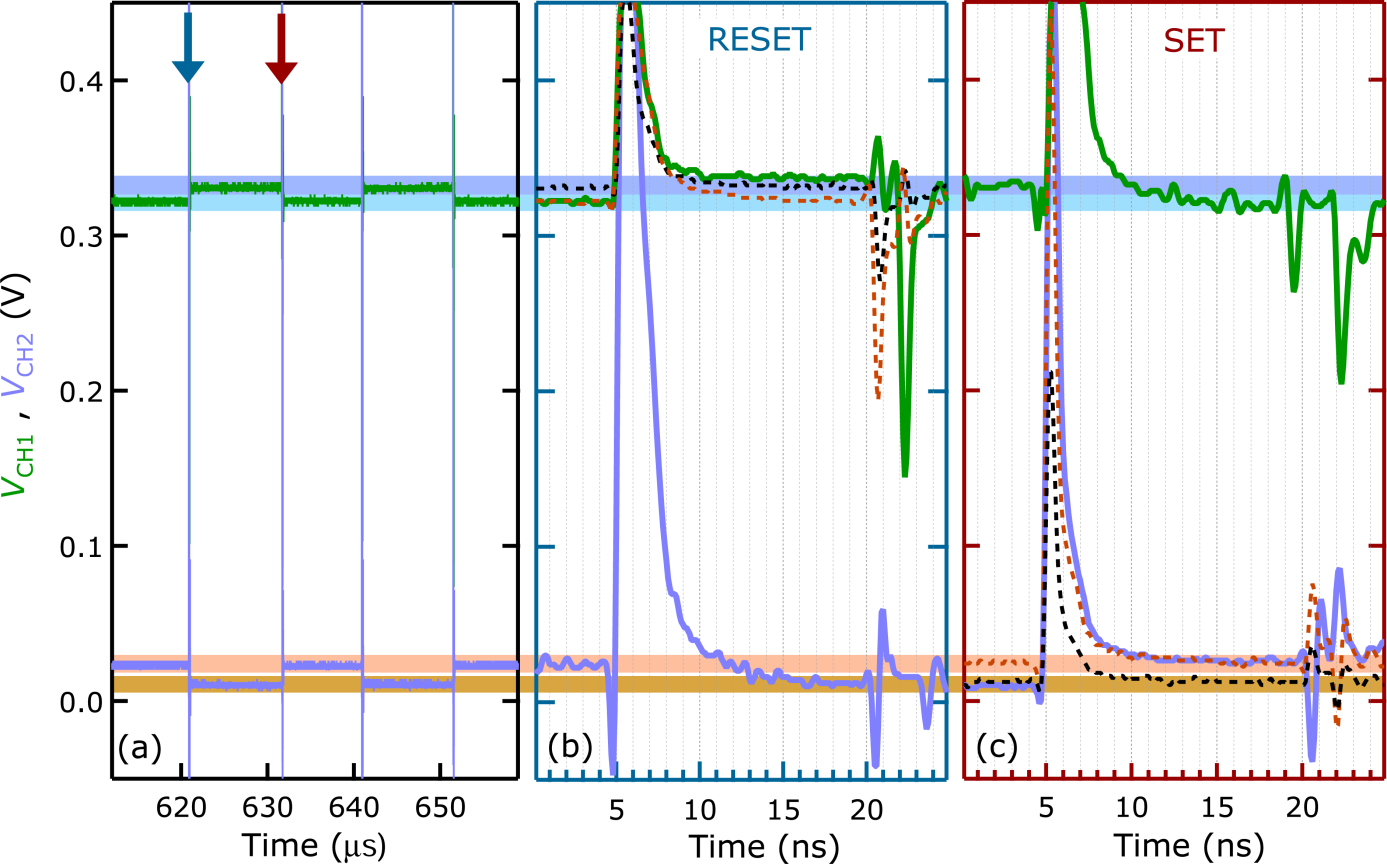
(a) Subsequent set and reset transitions triggered by the voltage pulses fired by G1 and G2, respectively, as seen by the voltage signals acquired at the CH1 and CH2 channels of the oscilloscope. A DC bias voltage of 0.5 V was added to monitor the actual resistance state of the nanojunction between the short pulses. The blue and red arrows label the individual reset and set transitions between *R*_ON_ = 325 ± 16 Ω and *R*_OFF_ = 790 ± 81 Ω states, which are magnified on the nanosecond timescale in panels (b) and (c), respectively. (b) Transmitted (green) and reflected (light blue) voltage signals acquired upon a reset transition of the nanojunction executed by a single voltage pulse of G2. (c) Transmitted (light blue) and reflected (green) voltage signals acquired upon a set transition of the nanojunction executed by a single voltage pulse of G1. The brown and black dashed traces in panels (b) and (c) were recorded under identical conditions in transmission while the nanojunction was replaced by 330 Ω (brown) and 810 Ω (black) resistors chosen in accordance with the ON and OFF state resistances of the nanojunction, respectively. The time axes of panels (b) and (c) are off-set for clarity.

Reproducible resistive switchings due to subsequent 500 ps set and reset voltage pulses are demonstrated by displaying the voltage signals directly acquired at CH1 and CH2 in Figure 5a.

The data recorded at a sampling rate of 10 Gs/s over 50 μs illustrates the long-term stability of the ON and OFF states between the switching events. The two adjacent reset and set transitions labeled by the blue and red vertical arrows are magnified on a nanosecond time scale in Figure 5b and Figure 5c, respectively. Note, that the traces in both panels are color-coded consistently with Figure 4a. Consequently, the green (light blue) line in Figure 5b corresponds to the transmitted (reflected) voltage signal arising from the memristor junction while G2 is fired to trigger a reset transition. In Figure 5c the scheme is reversed: G1 is fired to induce a set transition and the transmitted (reflected) signal is displayed in light blue (green). The steady-state resistances are determined in both cases as *R*_M_ = (*V*_CH1_/*V*_CH2_ − 1)·25 Ω. In order to achieve the best possible voltage resolution of the equilibrium values between the pulses, the peak values were kept out of range and were not recorded. These equilibrium levels are reached within 5 ns (8 ns) after the rising edge of the switching pulses as seen in the transmitted (reflected) signals, whereas higher-order reflections arising from the distant, passive circuit elements arrive with approx. 15 ns delay, in agreement with the length and propagation speed of the applied long coaxial lines.

Concerning the time scale of the set and reset switchings we underline that the above values only represent upper estimates. We argue that, according to the data shown in Figure 2, the approx. 2 ns long, lower-magnitude instrumental tail superimposed on the falling edge of the switching pulses illustrated in Figure 4c is insufficient to further modify the resistive state of the nanojunction, yet it hinders the unambiguous read-out of the equilibrium levels of the green and light blue traces in Figure 5b and Figure 5c. In order to quantify the actual switching speed of our memristive device more accurately, we compare its transmission curves to those obtained on equivalently biased smd resistors specified to the same resistance values as the measured ON and OFF state resistances of the Ag/AgI/PtIr nanojunction. The latter, non-switching traces are plotted by the brown (330 Ω) and black (810 Ω) dashed lines in Figure 5b and Figure 5c. As expected, the memristor transmissions coincide within experimental error with the corresponding resistor transmissions before and after the set and reset transitions, while the transitions of the memristor curves between the two resistor curves occur within the duration of the switching pulse. This is especially demonstrative along the SET transition, where the transmitted signal of the memristor junction (light blue line in Figure 5) clearly switches from the initial resistance state (black dashed line) to the final resistance state (brown dashed line) already within the rising edge of the pulse, i.e., the switching happens in less than 500 ps. According to the telegraph equations the actual voltage drop on the junction is 2*V*_Refl_. From this we estimate 50–100 pJ energy dissipation along the switching pulses, which is more than an order of magnitude smaller than the programming energy of a flash memory cell.

## Conclusion

We have investigated the resistive switching behavior of Ag/AgI/PtIr nanojunctions created in an STM arrangement and operated in the regime of metallic conductance. We showed that the latter exhibits highly linear *I*(*V*) relations all the way up to the switching threshold voltages, whereas the dynamical aspects of resistive switching comply to the voltage–time dilemma characteristic to various Ag-based filamentary resistive switches: A moderate linear increase in the magnitude of the driving voltage facilitates an exponential acceleration of the switching. The emergence of such multiple, coexisting physical time scales is a fundamental ingredient to non-volatile data processing. By utilizing the custom-designed pulsed microwave setup also described in this paper in detail, nanosecond-scale switching times were verified. We emphasize that by the simultaneous featuring of the four key properties, i.e., linearity, analogue tunability, non-volatility and high switching speed, AgI-based memristive nanojunctions offer a viable material platform for memristor crossbar networks optimized for performing fast and highly accurate vector–matrix operations.
